# A Painless Bump: A Case Report of Cemento-Ossifying Fibroma of the Anterior Maxilla

**DOI:** 10.7759/cureus.41799

**Published:** 2023-07-12

**Authors:** Divyadharshini V, Jayanth Kumar Vadivel, Dhanraj Ganapathy

**Affiliations:** 1 Oral Medicine and Radiology, Saveetha Dental College, Saveetha Institute of Medical and Technical Sciences, Chennai, IND; 2 Prosthodontics, Saveetha Dental College, Saveetha Institute of Medical and Technical Sciences, Chennai, IND

**Keywords:** benign tumor, odontogenic tumor, cone-beam computed tomography (cbct), painless swelling, cementum, maxilla, ossifying fibroma, fibro-osseous, alkaline phosphatase

## Abstract

Cemento-ossifying fibroma (COF) is a benign odontogenic neoplasm. It is considered an ossifying fibroma with traces of interspersed cementum fragments. Here we present a case report of the occurrence of COF in the maxillary anterior region of an elderly woman. A 61-year-old female reported with a painless, progressive, slow-growing swelling on the upper front jaw region for the past five years. A single, localized, swelling on the anterior region of the maxilla which was non-tender and bony hard in consistency. Radiological examination consisting of orthopantomagram (OPG) and cone-beam computed tomography (CBCT) revealed increased thickness of bone over frontal, parietal and maxilla with alteration of trabecular pattern - cotton wool/ground glass. Serum alkaline phosphatase level was found to be 865 U/l, however, serum calcium level and other routine blood investigations (hemogram) were within normal limits. The above radiological and laboratory findings are more in favour of primary bone pathology and with a biopsy later correlating with histopathological findings; it was diagnosed as COF. Under conscious sedation, surgical excision of the bony mass was done along with extraction of associated teeth. The patient is currently on regular follow-up and planning for a dental prosthesis is in progress.

## Introduction

Cemento-ossifying fibroma (COF) is a benign odontogenic neoplasm. It is an ossifying fibroma that shows the presence of cementum in it [1]. The global prevalence of COF is 3.1% of all oral tumors. The first case report of COF was reported in the year 1872 by Manzel [2,3]. The term ossifying fibroma is derived from the Latin word ‘ossis’ meaning bone and ‘fy’ to render or make a structure, so cumulatively it means to convert a tissue to bone-like hardness. In 1992, WHO classified it as a benign fibro-osseous lesion and made two tumor subtypes. The two variants were based on the cementum content in the tumor. They were designated as cementifying fibroma and cemento-ossifying fibroma respectively [2,3]. The lesion is classified as juvenile or adult based on the age at presentation [4].

The tumor has a probable origin from the cells of the periodontal ligament and this may account for the cementum tissue formation within the tumor [5]. The clinical features of the tumor are that of an asymptomatic swelling affecting women more commonly in the mandible. The probable differential that could be considered could be diseases of bone-like fibrous dysplasia and Paget's disease. The recurrence rate varies from 8-20%. The adult variant tends to present in the third to fourth decade of life. In contrast, the juvenile variant is seen in the first decade of life [6]. As a result of this overlap in diagnostic criteria, clinicians, radiologists, pathologists, and oral surgeons may get confused while attempting to diagnose and treat fibro-osseous lesions in the mandible or maxilla. As a consequence, perceptions of how best to treat these lesions might be subjective and varied.

Here we present a case report of the occurrence of COF in the maxillary anterior region of an elderly woman. Though reports of ossifying fibroma are already published, we could not find any literature correlating with alteration in serum biochemistry which was seen in our case. ​​Consensus-based Clinical Case Reporting (CARE) Guideline was followed [7]. Informed consent was obtained from the patient to use his intra-oral pictures and relevant investigations for the benefit of the scientific community.

## Case presentation

Patient information and clinical findings

**Figure 1:**
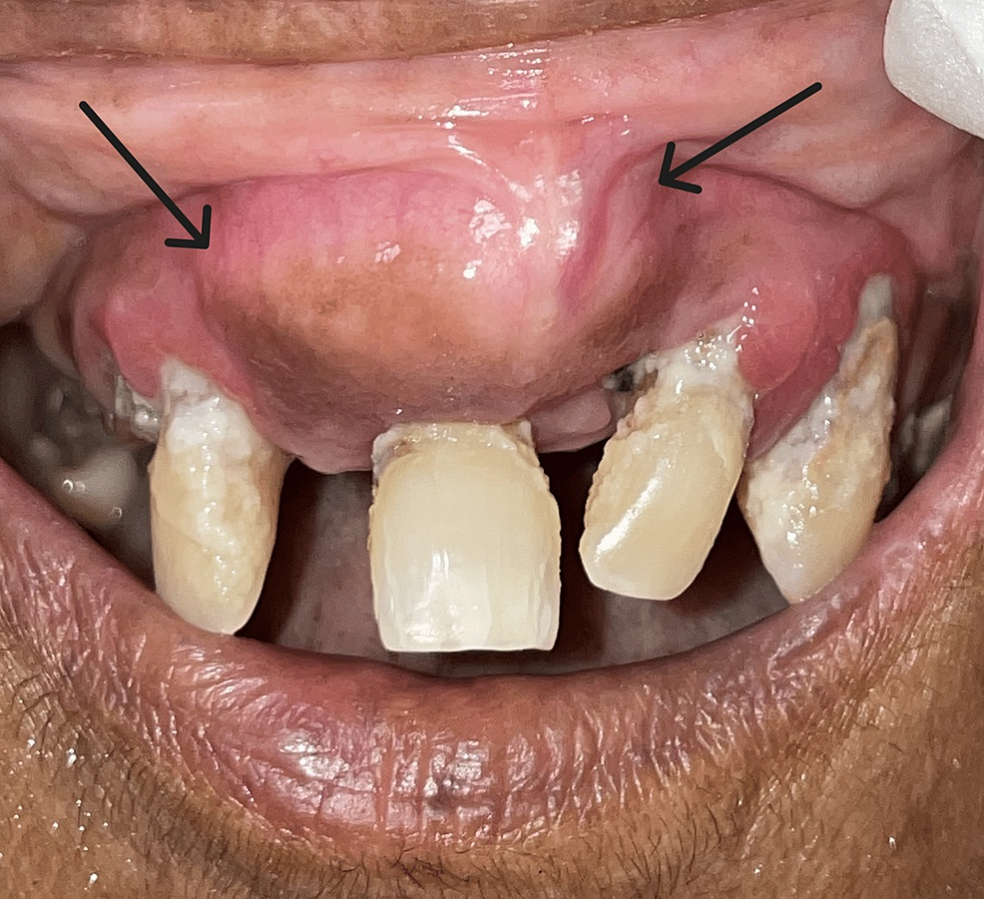
Intraoral photograph of a single localized swelling seen in the anterior region of maxilla (marked by black arrows)

A 61-year-old female reported with a painless, progressive, slow-growing swelling on the upper front jaw region for the past five years. A brief timeline of the way the swelling progressed is given in Table 1. On an extraoral examination, a single, ovoid localized swelling on the upper front region of the maxilla was noticed with a size of ∼2.2*2 cm. The skin over the swelling appeared normal in color. The inspection findings regarding size, shape, color, and extension were confirmed on palpation. The swelling was non-tender and bony hard in consistency, with no paraesthesia. On intraoral examination, a single, localized, oval-shaped swelling was seen in the upper anterior region, extending from the 12 to 22 teeth region. It measured about ∼3×2.5 cm, and the overlying mucosa was appears stretched with a greyish pigmentation appearance as seen in Figure 1. The swelling was smooth, firm in consistency, and non-tender on palpation. Oral hygiene was poor and severe periodontitis with root caries was observed. The upper anterior teeth adjoining the swelling were vital as identified by thermal testing and showed grade II mobility. The clinical differential diagnosis included swelling of the jaw with a bony origin like Paget's disease or benign tumor of the bone. As the teeth adjacent to the lesion was vital, a possibility of odontogenic inflammatory swelling was ruled out. 

**Table 1:** Clinical History Timeline

Duration prior to presentation	Complaint or feature as reported by the patient
5 years prior to presentation	Presence of a swelling which was slowly growing and progressive.
2 years prior to presentation	The swelling noted in the upper lip also
1 month prior to presentation	The swelling reached a point which led to incompetency of the lip.

Diagnostic assessment

Radiological Investigation and Findings

**Figure 2:**
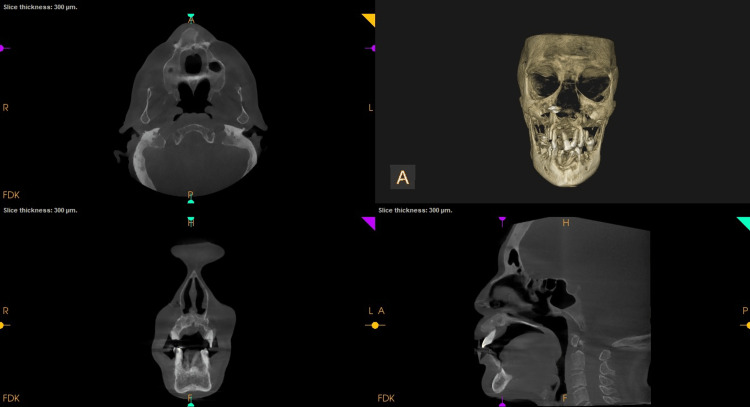
Preoperative cone-beam computed tomography (CBCT): Orthogonal slices in relation to 11. Axial and sagittal slice shows buccolingual expansion and 3D reconstruction showing the bony enlargement

**Figure 3:**
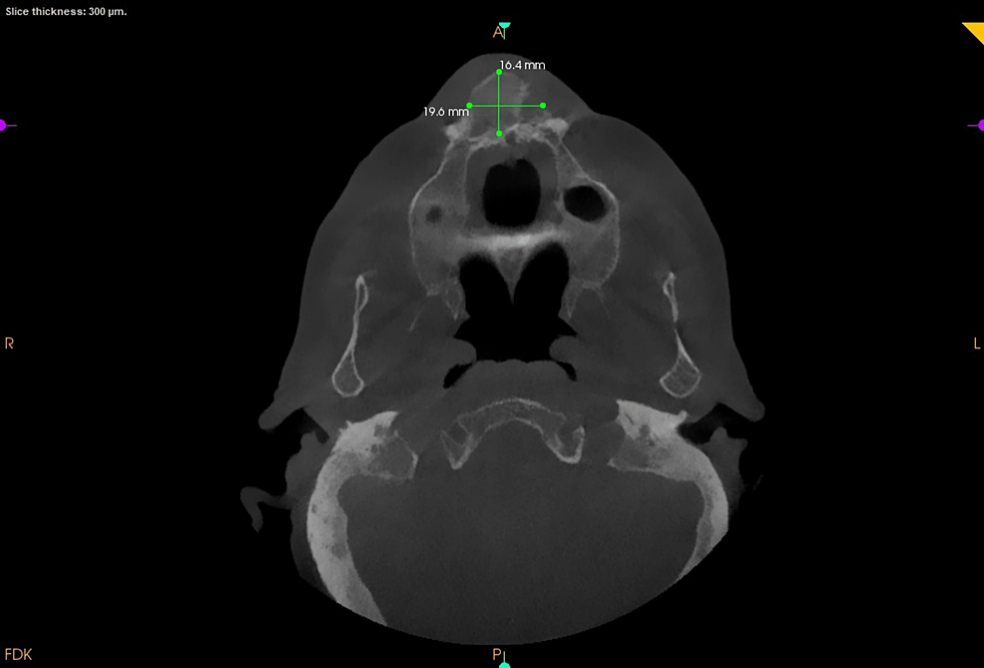
Axial view showing the buccolingual expansion of the cortical plates and the mesiodistal extent of the lesion

**Figure 4:**
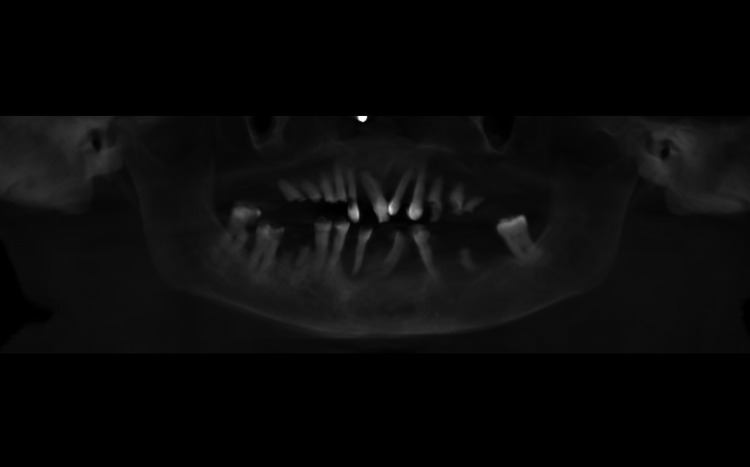
Preoperative cone-beam computed tomography (CBCT) images revealing the pathological migration of the teeth

An intraoral periapical radiograph of 11, 12, 13 showed coronal radiolucency involving enamel, dentin, and pulp in 12 with loss of crown tooth structure in 13. Following this a full skull cone-beam computed tomography (CBCT) of field of view (FOV) 16 X 17 cm was captured. The scan showed an increase in bone mass as evidenced by buccolingual widening of the alveolus in the maxilla (Figure 2). The buccolingual measurement at the widest section in the axial view was 16.4mm buccolinually and 19.6 mm mesiodistally (Figure 3). The trabeculae appeared more coarser and the cortical plate was marginally thickened. Tooth 11 showed interdental bone loss with root resorption. There was a generalised bone loss evident. There was no evident soft tissue capsule around or within the lesion. Teeth showed pathological migration in the anterior region of the frontal process of the maxilla (Figure 4). The displacement or pathological migration of 11 and 21 seen in the anterior region alone might be a pointer to denote that the epicentre of the lesion is the anterior maxilla.

Hematological investigations

On blood investigation it was found that the serum alkaline phosphatase level was 865 IU/L (Normal values are between 44-147 IU/L) with normal serum calcium level of 9.2g/dl (normal 9-11g/dl). Apart from this routine hemogram revealed hemoglobin 12.4g/dl (normal 12-14g/dl), red blood cell (RBC) count 4.73 million/cu.mm (normal 4.5-5.5 million/cu.mm) and white blood cell (WBC) count 9765/microlitre (normal 9000-11000/microlitre). Other blood investigations were within normal limits. All other hematological parameters were within normal range.

Excisional biopsy

**Figure 5:**
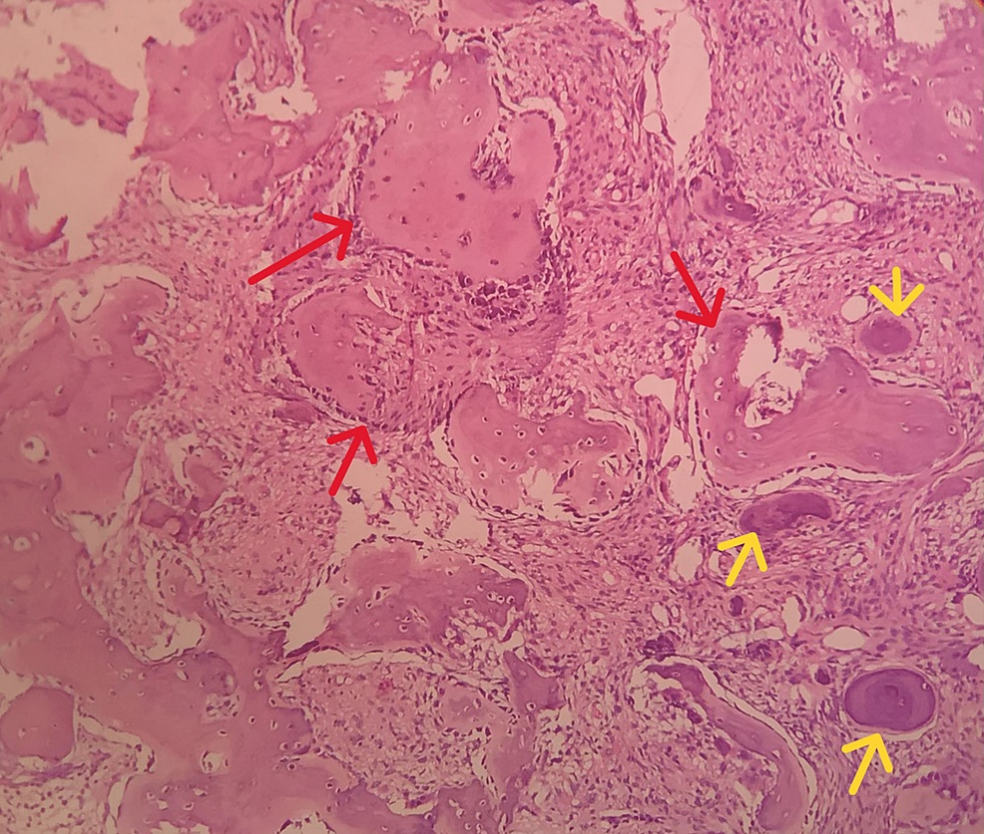
Low power photomicrograph of excisional biopsy specimen reveals presence of numerous irregular eosinophilic bony trabeculae (red arrows) with osteoblastic rimming and few areas showing osteoid formation (yellow arrows)

The patient was referred to oral surgery postgraduate clinic and an excisional biopsy was performed under conscious sedation. Preemptive 2% lignocaine without adrenaline was also administered through infraorbital nerve block and supplementary anterior and middle superior nerve blocks. A crevicular incision was placed over the 13 to 23 region and mucoperiosteal flap was elevated and excision and curettage of the mass were performed followed by thorough debridement. This was followed by extraction of teeth associated with the lesion (14, 13, 12, 24). Teeth that were infected root stumps (15, 16, 25, 26, 36, 46) were also extracted along with teeth with poor prognosis (47, 48). After achieving hemostasis the wound was closed with vicryl resorbable sutures. Postoperative antibiotics (amoxicillin 500 mg three times a day for five days and metronidazole 400 mg three times a day for five days), analgesics (paracetamol + aceclofenac three times a day for three days and two times a day for two days) and serratiopeptidase 5 mg two times a day for five days were prescribed. The patient was advised to be on a soft diet for five days and to do sponging with ice water from day two to day five of the postoperative phase. The biopsy findings were consistent and showed the presence of homogenous eosinophilic areas with scattered osteoblasts and irregular immature bone formation which were consistent with ossifying fibroma (Figure 5).

Follow-up and outcomes

**Figure 6:**
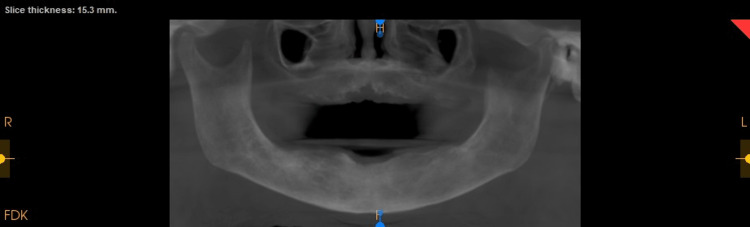
Postoperative cone-beam computed tomography (CBCT): Reconstructed Panoramic Image

**Figure 7:**
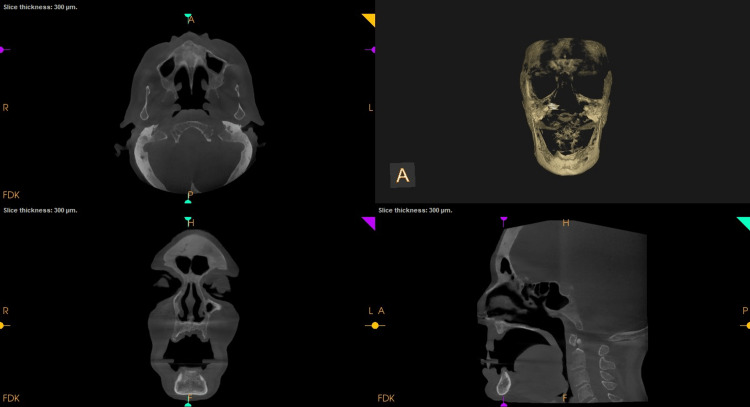
Postoperative cone-beam computed tomography (CBCT): Orthogonal slices in relation to 11 showing no bony changes that are evident

The patient was reviewed after one week for the occurrence of any pain and subsequently followed up for a period of two months for the occurrence of swelling. The postoperative pain was subminimal for the first three days and gradually dissipated. Postoperative healing was adequate and the patient exhibited no adverse manifestations till date and the mass has not shown any recurrence as seen in a postoperative scan taken after a period of two months (Figure 6, 7). The patient is presently undergoing prosthetic treatment for restoration of esthetics and function.

## Discussion

One of the most prevalent fibro-osseous lesions in the maxillofacial area is the ossifying fibroma, which often occurs in conjunction with fibrous dysplasia. They have distinct patterns of disease progression, but the various similarities in histomorphological and radiographic features pose difficulties in their classification and management. In the 2017 WHO classification, COF has been defined as a benign mesenchymal odontogenic tumor. This has been reaffirmed in the 2022 classification as well. Further in the 2022 classification it has been completely separated from the nonodontogenic juvenile, trabecular and psammatoid types [8].

In the clinical setting, COF appears as a painless, slowly expanding mass in the mandible rather than the maxilla, often located apical to posterior teeth, and more prevalent in females than males [9]. It is possible that the sole early clinical sign is the displacement of teeth, and the teeth in proximity to the lesion generally remain vital [10]. As a consequence, the patient typically fails to recognise the lesion until the growth has caused a notable change in the patient's facial appearance. The tumor can be clearly distinguished from the bone that surrounds it and will continue to grow, either slowly or vigorously, up to the point where it is surgically removed [11]. In our present report, the lesion was observed in the anterior maxillary region in a female patient in her 60s. The most common site of occurrence of the lesion is in the mandible. Displacement of teeth in the anterior maxilla was noted and the adjacent teeth were vital (as tested by thermal testing) and also the lamina dura was obscured. In our case also the tooth was vital which are the typical features of ossifying fibroma.

COF depends on the degree of mineralisation. It appears radiolucent in the initial stages; calcific flecks increase with maturity of the lesion and, finally, progress to a complete radiopaque mass [12]. A few studies have reported that a radiolucent pattern is more common in the younger age group whereas a mixed radiolucent-radiopaque appearance is seen in older patients [13]. The characteristic feature of COF is its centrifugal growth pattern, expanding equally in all directions thus appearing as a well-circumscribed round mass. This characteristic round shape was not present in our case and there was increased thickness of bone over frontal, parietal and maxilla (entire craniofacial skeleton) with obliteration of marrow spaces. Mixed radiolucency radiopacity in the trabeculae elicited a cotton wool appearance which was evident in our case too. Hence with radiological features the probability of Paget's disease became dominant but the histopathology ruled against it.

Histologically, ossifying fibroma is composed of cellular fibrous connective tissue containing varying amounts of woven bone, lamellar bone, osteoid, round cementoid calcifications and irregularly shaped bone trabeculae. Owing to the presence of this cementum-like material, ossifying fibromas have also been called COF [14]. The pattern of bone trabeculae in COF consists of woven bone surrounded by mature lamellar bone with osteoblastic rimming at the periphery. The cementum-like spherules demonstrate peripheral brush borders blending into the surrounding connective tissue. COF grows like a benign tumor. It can’t affect multiple bones. If a fibro-osseous lesion is affecting more than one bone and showing high serum alkaline phosphatase then the probability of fibrous dysplasia and Paget's disease have to be considered. However in this case the histological features were confirmative of COF.

In this case there was a 20-fold increase in the concentration of alkaline phosphatase. Alkaline phosphatase is a hydrolase enzyme with a role in dephosphorylation that is distributed primarily in the liver and bone which together account for 91%. An increase in alkaline phosphatase in the bone implies that there is an increase in the activity of osteoblasts. An increase in the concentration of this enzyme is noted in bone carcinomas and Paget's disease where there is an increase in bone turnover [15]. In a study of the alkaline phosphatase levels in 50 fibro-osseous lesions, which had 17 cases of ossifying fibroma in the age range of six to 65 years, there was only one patient who had an elevation of alkaline phosphatase [16]. However to date there could be no explanation for the elevation of this enzyme in the serum. Interestingly that case also involved multiple craniofacial bones like our case report. 

To date there are no studies in the literature which report an elevation of alkaline phosphatase in patients with cemento-ossifying fibroma. Normally an increase in alkaline phosphatase is seen in cases of lesions having an active osteoblastic activity. In case of Paget's disease, which is common in the current age and site of the case report, we anticipated to look for Paget's disease and hence had requested the test. But to our surprise we noted that there was an increase in alkaline phosphatase and histopathology showed ossifying fibroma.

The differential diagnosis of COF includes other fibro-osseous lesions, the most common benign fibrous dysplasia. Fibrous dysplasia is more common in the maxilla and it tends to grow longitudinally with ill-defined margins and is self-limiting. COF can be differentiated from Pindborg's tumor, as the latter is mostly associated with an impacted tooth and presents with flecks of calcification [17].

The treatment of COF varies according to the size of the lesion. The most recommended therapy for COF is curettage or enucleation. COF appears to be well demarcated from the surrounding bone and hence allows easy enucleation. Radical resection followed by bone grafting can be considered in cases of recurrence or aggressive nature of the lesion. However, in a study of 64 cases of COF a recurrence rate of 28% was reported in 18 patients treated by surgical curettage and/or enucleation [18], whereas Slootweg and Muller, in their study, revealed similar results with both conservative surgery and wider resection [19], recurrence was noted by Liu et al. to occur anywhere between six months and seven years following surgery [20]. Surgical resection was performed in our institution and long-term monitoring of these patients is thus recommended.

## Conclusions

Cemento-ossifying fibroma is a benign odontogenic tumor where some difficulty persisted regarding its pathogenesis. Here we have a case report of an elderly female who had presented with slow-growing swelling for several years causing facial disfigurement and which required a surgical excision. This was among the few cases which had reported an elevation of alkaline phosphatase in ossifiying fibroma. Interestingly both cases had involvement of multiple craniofacial bones. Though there was no recurrence noted in the patient to date an early presentation of the patient would have spared the needless extraction of multiple teeth in them. From this case report we can understand that there are instances of elevation of alkaline phosphatase in ossifying fibroma and it might be prudent to do a study of alkaline phosphatase levels in patients with ossifying fibroma to understand the clinical significance of the parameter. 
